# Construction of LncRNA-mediated CeRNA network for investigating the immune pathogenesis of myocardial infarction

**DOI:** 10.1097/MD.0000000000037413

**Published:** 2024-03-08

**Authors:** Dongmei Wei, Yuanting Meng, Hua Fan, Yang Sun, Rongtao Chen

**Affiliations:** a Cardiovascular Department, Liuzhou Traditional Chinese Medical Hospital, Liuzhou, China; b Guangxi University of Chinese Medicine, Nanning, China.

**Keywords:** ceRNA, immune-related, lncRNA, myocardial infarction

## Abstract

**Background:**

Myocardial infarction (MI) is a cardiovascular disease that seriously threatens human health. However, an immune-related competitive endogenous RNA (ceRNA) network has not been reported in MI.

**Methods:**

The GSE66360, GSE19339, GSE97320, GSE61741, and GSE168281 datasets were acquired from the Gene Expression Omnibus (GEO) database. The differentially expressed genes (DEGs) and differentially expressed miRNAs (DEmiRNAs) from MI patients and healthy controls were screened and an immune-related ceRNA network was constructed. Furthermore, the key long noncoding RNAs(lncRNAs) highly related to the immune mechanism of MI were identified utilizing the random walk with restart algorithm. Finally, the expression of the hub genes was further verified in the GSE66360, GSE19339, and GSE97320 datasets, and quantitative

real-time polymerase chain reaction (qRT-PCR) was performed for the MI patients and healthy controls.

**Results:**

A total of 184 differentially expressed immune-related genes

(DE-IRGs) and 432 DE-miRNAs were obtained, and an immune-related ceRNA network comprising 1421 lncRNAs, 61 DE-miRNAs, and 139 DE-IRGs was constructed. According to the order of stress, betweenness, and closeness, NEAT1, KCNQ1OT1, and XIST were identified as key lncRNAs. Moreover, random walk with restart analysis also suggested that NEAT1, KCNQ1OT1, and XIST are key lncRNAs. Subsequently, a ceRNA network of 10 hub genes and 3 lncRNAs was constructed. Finally, we found that the expression of FCER1G and TYROBP significantly differed between MI patients and control individuals in the GSE66360, GSE19339, and GSE97320 datasets. qRT–PCR revealed that the expression of NEAT1, KCNQ1OT1, XIST, FCER1G, and TYROBP was significantly elevated in MI tissue samples compared to healthy control tissue samples.

**Conclusion:**

NEAT1, KCNQ1OT1, XIST, FCER1G, and TYROBP are involved in MI and can be used as molecular biomarkers for the screening and diagnosis of MI. Furthermore, the immune system plays an essential role in the onset and progression of MI.

## 1. Introduction

Myocardial infarction (MI) is a severe clinical condition caused by myocardial degeneration or necrosis and has high mortality and morbidity rates despite substantial improvements diagnosis and treatment over the past decade.^[1]^ Due to the immediate reperfusion strategy and other medical therapies, the effectiveness of treatment of MI has been greatly improved. Nevertheless, challenges remain. Patients who develop cardiogenic shock still have a high 30-day mortality of at least 40%.^[2]^Rapid diagnosis can significantly reduce the mortality of MI. Currently, MI is usually diagnosed by observing changes via an electrocardiogram or assessing myocardial injury biomarkers, such as cardiac troponin I/T or the MB isoenzyme of creatine kinase. However, these diagnostic methods lack sufficient specificity and sensitivity, leading to misdiagnosis.^[3]^ Therefore, exploring novel diagnostic and prognostic biomarkers with high sensitivity and specificity, could pave the way for the ongoing discovery of new therapeutic targets for MI.^[4]^

Immune and inflammation-related genes and biological processes play a critical roles in heart damage and repair, and are considered markers of MI, as are the activation of innate and adaptive immune responses.^[5]^The results of a previous study indicated that immune mechanisms play a crucial role in MI. For example, the numbers of mast cells, M2 macrophages, and eosinophils in patients with MI affect the function of the heart,^[6]^ providing a new insights into the importance of immune regulation in MI. In addition, with the development of bioinformatics and genomics, the study of immune mechanisms can provide predictive value for the risk prognosis of diseases with potential benefits for patients.^[7]^

In recent years, multiple studies have confirmed that long noncoding RNAs (lncRNAs) exhibit dynamic changes in various cardiovascular diseases and participate in disease regulation through multiple molecular mechanisms, these changes may be important for the development and progression of MI.^[8]^ LncRNAs are defined as RNA transcripts longer than 200 base pairs and that are the main class of noncoding RNA.^[9]^ LncRNAs are involved in regulating disease progression and disease biological behavior by interacting with microRNAs (miRNAs) or messenger RNAs (mRNAs). LncRNAs containing miRNA response elements can compete with miRNA target genes to regulate miRNA expression, and such lncRNAs are known as competitive endogenous RNA (ceRNAs).^[10]^ CeRNA networks have become increasingly important in the development and progression of MI. For example, the lncRNA KLF3-AS1 ameliorates cardiomyocyte scorching and MI through the miR-138-5p/Sirt1 axis^[11]^; the lncRNA TUG1, a competitive endogenous RNA, mediates CTGF expression through miR-133b in myocardial fibrosis after MI onset^[12]^; and the construction of lncRNA-associated ceRNA networks in MI can identify functional lncRNAs. As such, the critical lncRNAs can be effectively identified and analyzed based on the lncRNA-associated ceRNA network.^[13]^ LncRNAs are widely involved in metabolism, immunity and other crucial physiological processes, and are closely related to the occurrence and development of cardiovascular diseases and other diseases; therefore the functional characterization of lncRNAs may be helpful in the prevention, monitoring and treatment of diseases.^[14]^In addition, lncRNA biomarkers may be involved in inflammatory and immune-related biological processes in MI.^[6]^ Yin et al reported that the lncRNA SNHG12 upregulated SIRT1 by targeting miR-199a, activating the AMPK pathway, reversing the damage caused by miR-199a to cerebral microvascular endothelial cells, and improving the inflammatory response.^[15]^ These results provides new insights into the mechanisms of cardiovascular disease and immune regulation.

However, immune-related ceRNA networks mediated by lncRNAs have not been reported in MI. Therefore, we focused on identifying key mRNAs and lncRNAs involved in MI by constructing lncRNA-mediated immune-related ceRNA networks to provide a reference for exploring the potential molecular mechanisms involved in MI.

## 2. Materials and methods

### 2.1. Data sources

The transcriptome sequencing data and sample information of the GSE66360, GSE19339, GSE97320, GSE61741, and GSE168281 datasets were downloaded from the Gene Expression Omnibus (https://www.ncbi.nlm.nih.gov/geo/) database. The GSE66360 dataset was used for differential expression analysis (49 MI and 50 control samples); the GSE19339 dataset (4 MI and 4 normal samples) and GSE97320 dataset (3 MI and 3 control samples) were used to verify the expression of the genes; the GSE61741 dataset was used for differential miRNA expression analysis (62 MI and 94 control samples); and the GSE168281 dataset was used for selecting the lncRNAs with expression data (3 MI samples). In addition, a total of 1793 immune-related genes (IRGs) were acquired from the ImmPort (https://www.immport.org/home) database, and 3279 IRGs were acquired from the AmiGO (http://amigo.geneontology.org/amigo/search/bioentity) database. Finally, 4004 IRGs were obtained by integrating the IRGs obtained from the above 2 databases (Table S1, Supplemental Digital Content, http://links.lww.com/MD/L871).

### 2.2. Differential expression analysis

The limma package (version 3.46.0)^[16]^ was applied to select the differentially expressed genes (DEGs) and differentially expressed miRNAs (DEmiRNAs) between the MI and control samples in the GSE66360 and GSE61741 datasets with the following filters: (*P* < .05, and |log2fold change| ≥ 1.0) (MI vs control). For differential expression analysis, this study did not filter the samples before statistical analysis; rather, we standardized only the data, and eliminated some low-quality genes. The VennDiagram package (version 1.6.20)^[17]^ was used to determine the interactions between the DEGs and IRGs that were differentially expressed IRGs (DE-IRGs).

### 2.3. Topological structure and subnetwork screening of the ceRNA network

The Starbase database was used to predict the relationships between lncRNAs and DEmiRNAs, and the predicted lncRNAs were intersected with those expressed in the GSE168281 database. The miRwalk database was used to predict DE-IRGs targeted by DEmiRNAs, and the predicted mRNAs were intersected with DE-IRGs. Subsequently, the relationships of lncRNAs, miRNAs, and mRNAs were input into the Cytoscape (version 3.8.2)^[18]^ to construct the lncRNA-miRNA-mRNA network. The CytoHubba tool of the Cytoscape and the Analyse Network tool were used to analyze the topological structure of the lncRNA-miRNA-mRNA network.

### 2.4. Functional enrichment analysis of ceRNA networks and target genes

The clusterProfiler package (version 3.18.0)^[19]^ and FunRich software were used to perform the Gene Ontology (GO) and the Kyoto Encyclopedia of Genes and Genomes (KEGG) enrichment analyses of the target genes. Moreover, the enrichplot package (version 1.10.2) and the ggplot2 package (version 3.3.3) were applied to visualize the enrichment results.

### 2.5. PPI network of target genes

The Search Tool for the Retrieval of Interacting Genes (STRING) Database (https://www.string-db.org/) was used to construct protein–protein interaction (PPI) networks for target genes with a confidence equal to 0.6. The discrete proteins were subsequently removed, after which the interaction relationships were calculated. The Network tool of the Cytoscape (version 3.8.2)^[18]^ was subsequently used to analyze the network. To ensure the reliability of the results, we defined the top 10 genes were regarded as hub genes.

### 2.6. Screening of key lncRNAs by Random Walk Restart

The RandomWalkRestartMH package (version 1.10.0)^[20]^ was used to obtain the top 15 nodes according to the relative ranking of the hub genes with the random walk with restart algorithm. The most frequently occurring lncRNAs were selected as key lncRNAs.

### 2.7. Expression verification of the hub genes

The expression of hub genes in the MI samples and normal samples was verified in the GSE66360, GSE19339, and GSE97320 datasets. The ggplot2 (version 3.3.3), Wilcoxon test, and ggpubr (version 0.4.0) packages were used to construct boxplots for visualization.

### 2.8. qRT-PCR

The expression of NEAT1, KCNQ1OT1, XIST, FCER1G, and TYROBP was verified in the 10 normal and 10 AMI samples. These samples were collected from the Liuzhou Traditional Chinese Medical Hospital, and all patients signed informed consent forms. This study was approved by the Medical Ethics Committee of Liuzhou Traditional Chinese Medical Hospital (approval number: 2022JAN-KY-YN-009-01). Total RNA of samples was extracted from the samples with TRIzol reagent (Thermo Fisher, Shanghai, CN). Then, reverse transcription was performed using the sweScript RT First-Strand cDNA Synthesis All-in-OneTM First-Strand cDNA Synthesis Kit (Servicebio, Wuhan, CN). Moreover, 2x Universal Blue SYBR Green qPCR Master Mix (Servicebio) was used for PCR. The primers used are shown in Table 1.

**Table 1:** Primers and their sequences for RTPCR analysis.

Primer	Sequence (5′–3′)
KCNQ1OT1 F	GTGTAGGGAGGAGAGCAAGGAT
KCNQ1OT1 R	GGAGGACAGTGGTCAGAGAGGT
NEAT1 F	GCTGTTGGAGTCGGTATTGTTG
NEAT1 R	ATCTGCTCGCCATGAGGAAC
XIST F	GCTAATCTACTTGGATGGGTTGC
XIST R	GTTGTTGCCCAGTGGTAGTGA
TYROBP F	GTAAGTGGTCTCCGTCCTGTCC
TYROBP R	GATAAGGCGACTCGGTCTCAGT
FCER1G F	CCTGGATGCCATCCTGTTTC
FCER1G R	CTCCTGGTTCCTGGTGCTCA
GAPDH F	GGAAGGTGAAGGTCGGAGT
GAPDH R	TGAGGTCAATGAAGGGGTC

## 3. Results

### 3.1. Differential expression analysis

**Figure 1.**
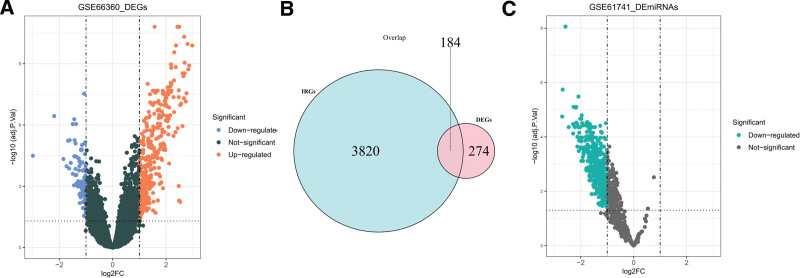
Identification of DEGs, DE-miRNAs and DE-IRGs in MI. The horizontal coordinate logFC indicates the multiplicity of the difference (MI/normal), and the vertical coordinate indicates the confidence level-log10 (*P* value). Each point in the graph represents a gene, and the dashed lines on the horizontal and vertical axes indicate the logFC absolute threshold of 1 and the *P* value threshold of .05, respectively. A. Volcano plot of DEGs. The blue and orange dots represent significant DEGs, with orange dots indicating that the gene expression was upregulated in MI samples, and blue dots indicating that the gene was downregulated in MI samples. B. Venn diagram of IRGs and DEGs. The blue and pink circles indicate IRGs and DEGs, respectively, and their intersection represents overlapping genes (i.e., DE-IRGs). C. Volcano plots of DEmiRNAs. The green dots represent significantly downregulated DEmiRNAs in the MI samples. DEGs = the differentially expressed genes, DEmiRNAs = differentially expressed miRNAs, IRGs = immune-related genes, MI = myocardial infarction.

In the GSE66360 dataset, 458 DEGs were acquired between the MI and control samples, including 344 upregulated DEGs and 114 downregulated DEGs, were identified (Fig. 1A). Furthermore, 184 DE-IRGs were obtained for further analysis (Fig. 1B). In the GSE61741 dataset, 432 DE-miRNAs were acquired between the MI and normal samples. These DE-miRNAs were all downregulated (Fig. 1C).

### 3.2. Topological structure and subnetwork screening of the ceRNA network

**Figure 2.**
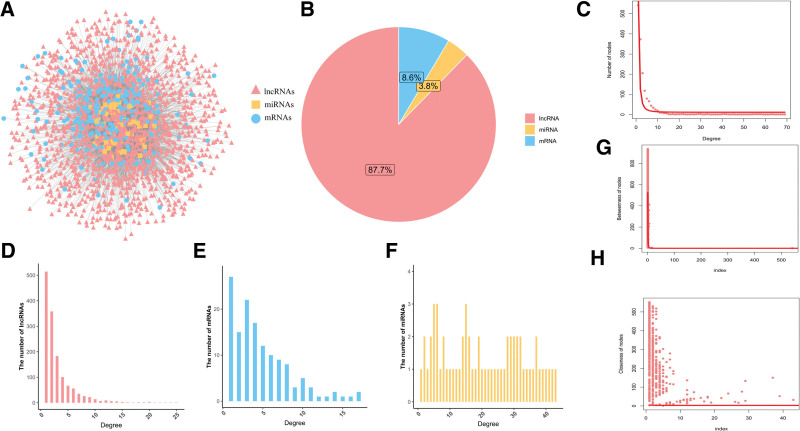
Construction and analysis of the ceRNA network. A. CeRNA network. The blue, yellow, and red nodes represent DE-IRGs, miRNAs, and lncRNAs, respectively. Lines represent interactions among DE-IRGs, miRNAs, and lncRNAs. B. Pie chart of the number of IRGs, miRNAs, and lncRNAs in the network. C. Degree distribution of all nodes in the ceRNA network. D. Degree distribution of the DE-lncRNAs. E. Degree distribution of the DE-IRGs. F. Degree distribution of the DE-miRNAs. G. Node betweenness in the ceRNA network. H. Node closeness in the ceRNA network. ceRNA = competitive endogenous RNA, DEGs = the differentially expressed genes, DEmiRNAs = differentially expressed miRNAs, lncRNAs = long noncoding RNAs.

**Figure 3.**
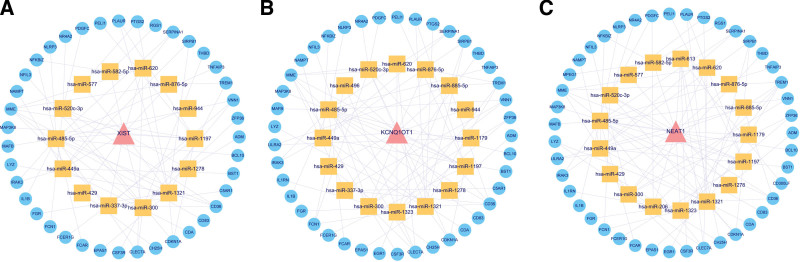
Analysis of immune-associated lncRNAs from the ceRNA network identified with the random walk approach. A. Subnetwork of the lncRNA NEAT1, B. Subnetworks of the lncRNA KCNQ1OT1, and C. Subnetworks of the lncRNA XIST. ceRNA = competitive endogenous RNA, lncRNAs = long noncoding RNAs.

A total of 1421 lncRNAs were obtained from the Starbase database and GSE168281 dataset. In addition, 61 DEmiRNAs and 139 DE-IRGs were obtained from the ceRNA network. Among the 1421 lncRNAs (87.7%), 61 DEmiRNAs (3.8%), and 139 DE-IRGs (8.6%), and 5093 relationship pairs were predicted (Fig. 2A and B). The degree of ceRNA, lncRNA, mRNA, and miRNA enrichment indicated that the network conformed to the scale-free distribution network (Fig. 2C–F), and the betweenness and closeness of nodes in the ceRNA network are shown in Figure 2G and H. Finally, the stress, betweenness, and closeness of NEAT1, KCNQ1OT1, XIST, hsa-miR-485-5p, hsa-miR-665, hsa-miR-520c-3p and hsa-miR-1321 identified them as the top 20 of the nodes (Table 2). Among the top 20 nodes, NEAT1, KCNQ1OT1, and XIST were used to construct the ceRNA network. XIST was linked to 14 miRNAs and 39 mRNAs (Fig. 3A); KCNQ1OT1 was linked to 16 miRNAs and 41 mRNAs (Fig. 3B); and NEAT1 was linked to 17 miRNAs and 43 mRNAs (Fig. 3C).

**Table 2:** Closeness, betweenness, and stress of the top 20 nodes in the ceRNA network.

Closeness	node_name1	Betweenness	node_name2	Stress	node_name3
815.83333	NEAT1	325457.7451	hsa-miR-485-5p	2.91E + 07	hsa-miR-485-5p
806.75	KCNQ1OT1	272258.4575	hsa-miR-665	2.61E + 07	hsa-miR-665
787.66667	XIST	199563.4753	hsa-miR-520c-3p	1.53E + 07	hsa-miR-1321
723.16667	OIP5-AS1	160738.4917	hsa-miR-330-5p	1.51E + 07	hsa-miR-520c-3p
708.66667	hsa-miR-485-5p	157891.561	NEAT1	1.33E + 07	hsa-miR-330-5p
702.08333	HCG18	154667.0335	hsa-miR-1321	1.28E + 07	hsa-miR-330-3p
701.33333	GABPB1-AS1	143517.0601	hsa-miR-330-3p	1.18E + 07	NEAT1
698.66667	MALAT1	140483.1325	hsa-miR-513a-5p	1.07E + 07	hsa-miR-513a-5p
696.08333	NUTM2B-AS1	138916.7549	KCNQ1OT1	1.05E + 07	KCNQ1OT1
696	hsa-miR-665	133570.4009	hsa-miR-671-5p	1.02E + 07	hsa-miR-525-5p
693.25	AL157392.3	132622.5501	hsa-miR-525-5p	9800952	hsa-miR-671-5p
676.66667	AC239868.1	132009.0966	hsa-miR-944	9195278	hsa-miR-944
674.5	MIR29B2CHG	119045.0203	hsa-miR-515-5p	8709832	XIST
670.83333	LIF	108881.2094	XIST	8501328	hsa-miR-455-3p
666.58333	TREM1	105611.7789	hsa-miR-1323	8083928	hsa-miR-515-5p
662.58333	SNHG16	103251.47	hsa-miR-455-3p	7895914	hsa-miR-577
656.91667	AC068888.1	102151.6171	hsa-miR-577	7536324	hsa-miR-34c-5p
656.91667	SIRPB1	95542.99293	hsa-miR-296-3p	7498230	hsa-miR-519c-3p
656	hsa-miR-520c-3p	93733.91193	hsa-miR-620	7360802	hsa-miR-519b-3p
652	hsa-miR-1321	92375.38358	hsa-miR-543	7310176	hsa-miR-490-3p

### 3.3. Functional enrichment analysis of ceRNA networks and target genes

**Figure 4.**
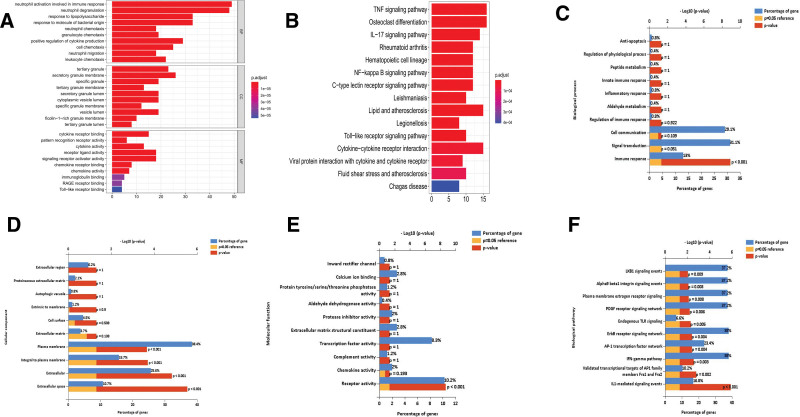
GO functional and KEGG pathway enrichment analyses of target genes involved in MI. The x-axis shows the number of genes, and the y-axis shows the GO and KEGG pathway terms. The −log10 (*P* value) of each term is coloured according to the legend. A. GO enrichment. B. KEGG pathway. C. Biological process. D. Cytological component. E. Molecular function. F. KEGG pathway enrichment analysis with FunRich. GO = gene ontology, KEGG = Kyoto Encyclopedia of Genes and Genomes, MI = myocardial infarction.

To explore the functions of the targeted genes, we performed GO and KEGG enrichment analyses. Among the enriched GO terms, 757 biological process (BP) terms, 28 cellular component (CC) terms, and 29 molecular function (MF) terms were enriched for the target genes; these included neutrophil activation involved in the immune response, tertiary granule, and cytokine receptor binding (Fig. 4A). According to the KEGG enrichment results, 48 KEGG pathways were enriched for the target genes, including the TNF signaling pathway, osteoclast differentiation, lipids, and atherosclerosis (Fig. 4B). Moreover, the FunRich results indicated that the target genes were significantly correlated with the immune response (*P* < .001) according to the GO-BP terms (Fig. 4C); significantly correlated with the plasma membrane (*P* < .001), integral to the plasma membrane (*P* < .001), extracellular (*P* < .001) and extracellular space (*P* < .001) according to the GO-CC terms (Fig. 4D); and significantly correlated with receptor activity (*P* < .001) according to the GO-MF terms (Fig. 4E). According to the KEGG enrichment results, the target genes were significantly correlated with the ErbB receptor signaling network (*P* = .004), the AP-1 transcription factor network (*P* = .004), and the IFN-gamma pathway (*P* = .003), validating the transcriptional targets of the AP1 family members Fra1 and Fra2 (*P* = .002) and revealing IL-mediated signaling events (*P* < .001) (Fig. 4F).

### 3.4. PPI network of target genes

**Figure 5.**
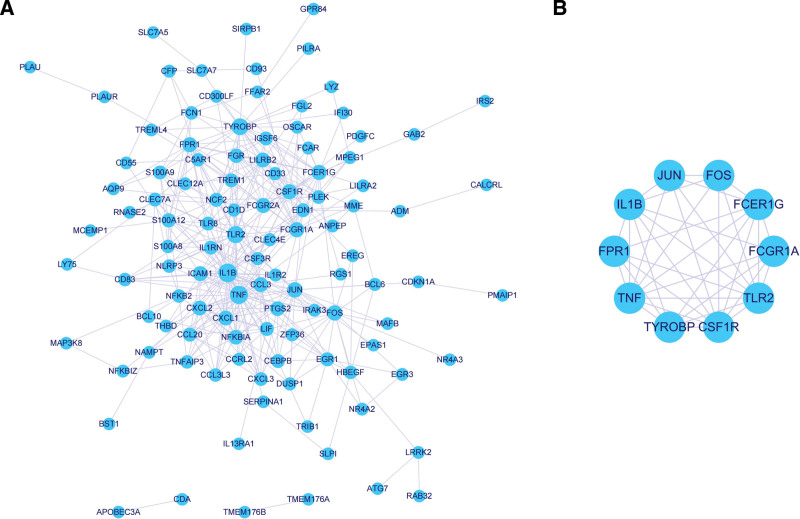
A. PPI network of DE-IRGs in the ceRNA network. Circles represent DE-IRGs, and the size of the node represents the degree. Lines represent interactions between proteins encoded by DE-IRGs, and the width of the line represents the combined score between DE-IRGs. B. PPI network of the top 10 target genes. ceRNA = competitive endogenous RNA, DEGs = the differentially expressed genes, PPI = protein–protein interaction.

To explore the interactions between the 139 target genes, we generated a PPI network with a confidence of 0.6. The PPI network contained 111 nodes and 742 edges (Fig. 5A), and the top 10 target genes were IL1B, TNF, TLR2, TYROBP, JUN, FOS, FCGR1A, FCER1G, CSF1R, and FPR1 (Table 3; Fig. 5B). These 10 target genes were regarded as hub genes for the subsequent analysis.

**Table 3:** The top 10 target genes of degree.

Symbol	Betweenness centrality	Closeness centrality	Degree	Stress
IL1B	0.205690927	0.543589744	78	11702
TNF	0.159544549	0.522167488	70	9002
TLR2	0.100654059	0.507177033	58	6140
TYROBP	0.14484918	0.447257384	54	7794
JUN	0.098938642	0.464912281	38	4484
FOS	0.099154446	0.449152542	36	4524
FCGR1A	0.041394894	0.449152542	34	2868
FCER1G	0.095383356	0.441666667	34	4398
CSF1R	0.084679533	0.454935622	34	4412
FPR1	0.060451104	0.415686275	32	3684

### 3.5. Screening of key lncRNAs via the random walk with restart analysis

**Figure 6.**
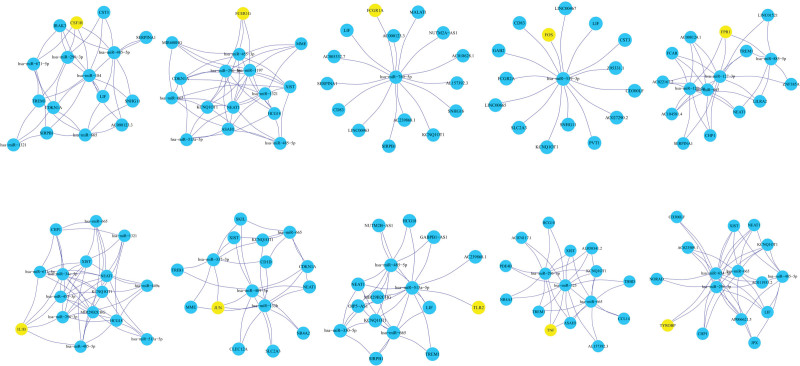
Random walk with restart analysis of the top 15 hub gene-related genes, yellow represents the hub genes, and blue represents the top 15 ranked nodes.

**Figure 7.**
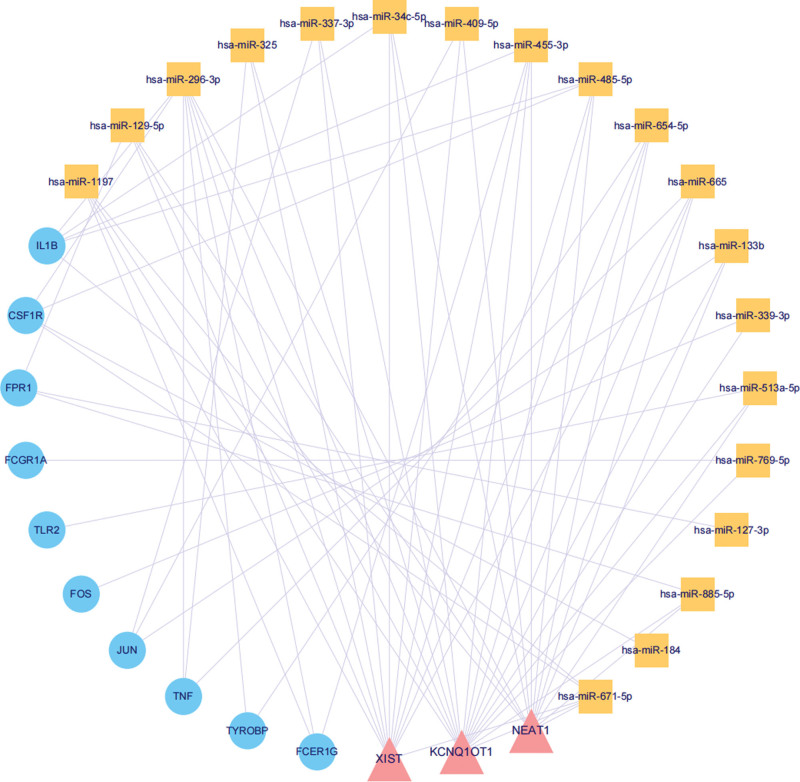
ceRNA network of 10 target genes and 3 lncRNAs. The pink triangles represent lncRNAs, the yellow squares represent miRNAs, and the blue circles represent hub genes. ceRNA = competitive endogenous RNA, lncRNAs = long noncoding RNAs.

The 10 hub genes of the PPI network were subjected to a random walk with restart analysis. The top 15 genes related to each hub gene identified via random walk with restart analysis were used to construct the ceRNA network. Among the 10 ceRNA networks, the top 3 most frequently occurring molecules were KCNQ1OT1, NEAT1, and XIST; these results are consistent with those in Figure 3 (Fig. 6). Subsequently, a ceRNA network of 10 target genes and 3 lncRNAs was constructed, and this network contained 19 miRNAs (Fig. 7).

### 3.6. Expression verification of the hub genes

**Figure 8.**
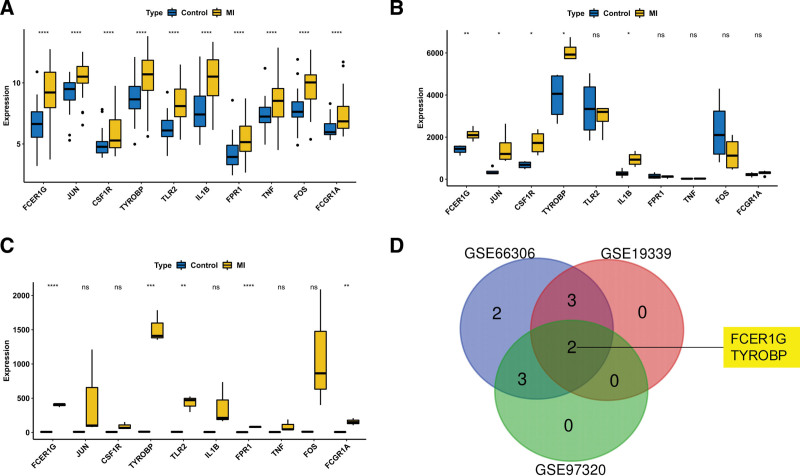
Expression and its validation of the hub genes. Yellow represents the MI samples, and blue represents the normal samples. *P < .05, **P < .01, ***P < .001, and ****P < .0001. A. Hub genes expressed in GSE66360. B. Hub genes expressed in GSE19339. C. Hub genes expressed in GSE97320. D. Genes were screened using a Venn diagram. MI = myocardial infarction.

To verify the expression of the 10 hub genes, we performed differential expression analysis on MI samples and normal samples in the GSE66360, GSE19339, and GSE97320 datasets. FCER1G and TYROBP were significantly different between the MI and normal samples in the above datasets (Fig. 8).

### 3.7. qRT-PCR

**Figure 9.**
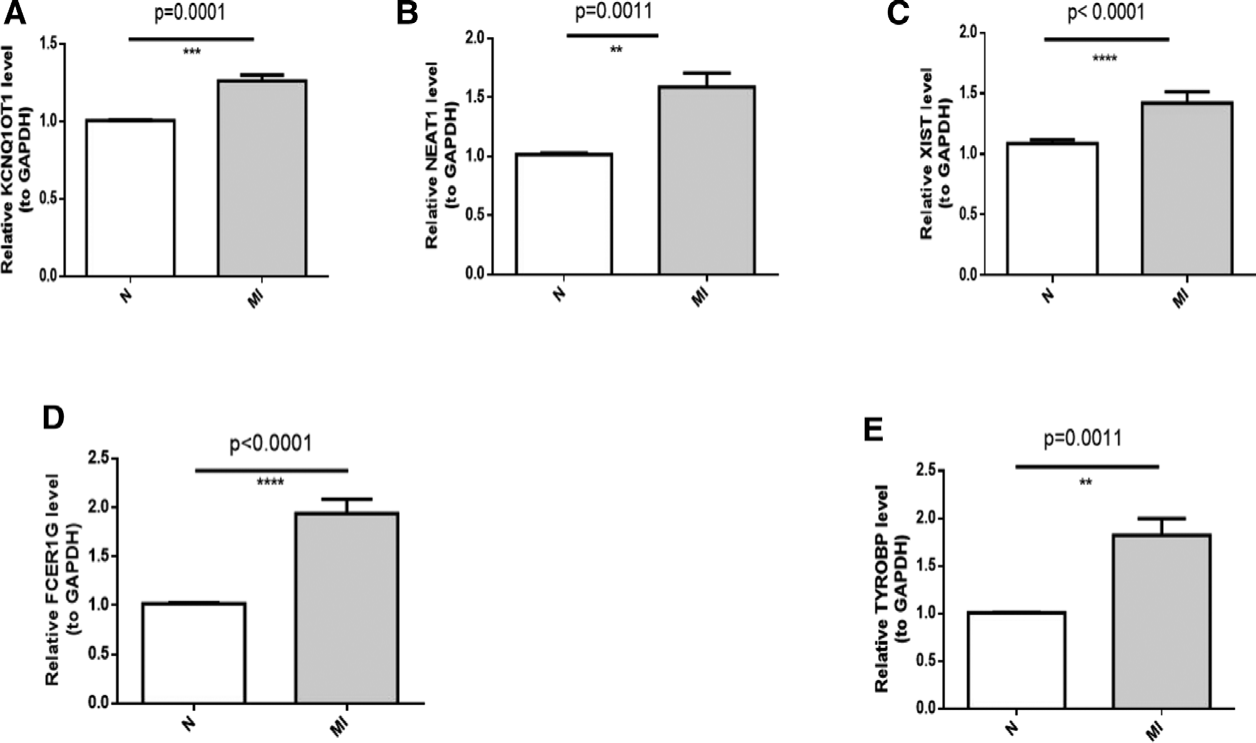
qRT-PCR validation of the key lncRNAs and hub IRGs of the ceRNA network. A. The relative expression of KCNQ1OT1 between MI patients and control individuals. B. The relative expression of NEAT1 between MI patients and control individuals. C. The relative expression of XIST between MI patients and control individuals. D. The relative expression of FCER1G between MI patients and control individuals. E. Relative expression of TYROBP between MI patients and control individuals. ceRNA = competitive endogenous RNA, lncRNAs = long noncoding RNAs, MI = myocardial infarction, qRT-PCR = quantitative Real-Time Polymerase Chain Reaction.

The results of qRT-PCR indicated that the expression levels of NEAT1, KCNQ1OT1, XIST, FCER1G, and TYROBP were significantly different between the normal and MI samples. The expression levels of NEAT1, KCNQ1OT1, XIST, FCER1G, and TYROBP were greater in the MI samples than that in the normal samples (Fig. 9). Among them, the expression patterns of FCER1G and TYROBP determined via qRT-PCR were consistent with the results of bioinformatics analysis.

## 4. Discussion

Increasing evidence illuminates that both the occurrence and development of MI and the repair after myocardial necrosis are closely associated with the immune-inflammatory response.^[21–24]^ Several studies have shown that lncRNAs involved in the inflammatory process may be a potential diagnostic and prognostic markers and therapeutic targets in MI.^[25,26]^ The lncRNA-mediated ceRNA regulatory network can provide new perspectives on the molecular basis of various diseases,^[27,28]^ and constructing a ceRNA network of immune-related lncRNAs can help elucidate the immune mechanisms of MI. In our study, we used integrative and computational methods to validate the expression profiles and interactions of mRNAs, miRNAs, and lncRNAs to explore the immune-related ceRNA network in MI.

In this study, we constructed a lncRNA-mediated immune-related ceRNA network based on the ceRNA theory. According to the constructed network, lncRNAs are involved in the immune response or inflammation in various diseases. For example, lipid metabolic disorders and inflammatory responses can be attenuated by KCNQ1OT1 knockdown or overexpression of miR-145-5p. KCNQ1OT1 acts as a molecular sponge for miR-145-5p and downregulate the expression of miR-145-5p.^[29]^ NEAT1 promotes the onset of rheumatoid arthritis by regulating the miR-144-3p/ROCK2 axis.^[30]^ The lncRNA XIST is highly expressed in psoriasis patients and is positively correlated with neutrophilic inflammation and psoriatic disease severity. XIST can be silenced by sponging miR-338-5p, which inhibits cell proliferation and inflammation.^[31]^ Therefore, the regulation of lncRNAs is a common phenomenon in various diseases from the perspective of the ceRNA network and will provide an important basis for the study of the molecular immune mechanisms of MI.

We constructed an immune-related ceRNA network in MI by mapping DEIRGs, DEmiRNAs, and DElncRNAs to the global network and analyzed the function of the target genes regulated by lncRNAs. The results of the GO analysis showed that target genes were significantly associated with immune-related biological processes, including leukocyte chemotaxis, neutrophilmigration, cellchemotaxis, positive regulation of cytokine production, granulocyte chemotaxis, neutrophil-chemotaxis, response to molecules of bacterial origin, response to lipopolysaccharide, neutrophil degranulation and neutrophil activation involved in production. Similarly, KEGG pathway analysis of these target genes revealed several enriched immune-related pathways, such as the TNF signaling pathway, bacterial infection, IL-17 signaling pathway, and hematopoietic cell line. The above results suggest that MI is closely associated with several important immune or inflammatory biological pathways, which is consistent with previous findings.^[32]^Therefore, these lncRNAs may play a pivotal role in the immune mechanisms in MI.

We applied the random walk with restart algorithm to the ceRNA network and ultimately identified the top 3 lncRNAs that are closely related to immune inflammation after MI. The 3 identified lncRNAs (NEAT1, KCNQ1OT1, and XIST) were consistent with the results for stress, betweenness, and closeness. The qRT-PCR results qRT-PCRs showed that the expression levels of NEAT1, KCNQ1OT1, XIST, FCER1G, and TYROBP in patients with MI were significantly greater than those in healthy individuals, and these results are consistent with the results of previous literature.^[5,33–35]^ Our study further supports the functional studies of lncRNAs in MI and provides a new therapeutic target for the treatment of MI.

As an immune-related gene, FCER1G mainly mediates the inflammatory pathway involved in mast cell allergy and can also transduce and activate a variety of immune receptor signals, thereby playing a role in mediating neutrophil and platelet activation. Previous studies have shown that the expression of FCER1G is significantly increased in MI patients and MI mouse models, and the area under the ROC curve (AUC) of FCER1G in the diagnosis of MI is 80.7%.^[36]^ TYROBP is believed to play an important role in the onset and development of Alzheimer’s disease,^[37]^ but genomic studies have shown that TYROBP is often highly expressed in cardiovascular diseases such as coronary artery disease,^[38]^ atrial fibrillation,^[39]^ and hypertrophic obstructive cardiomyopathy.^[40]^ TYROBP is a membrane-encoded immune-related signal transduction adaptin that is a key regulator of the immune system. TREM-1 associates with TYROBP to initiate an intracellular signaling cascade, leading to an expansion of the inflammatory response.^[41]^ Differential gene analysis was carried out on the gene chips of NAFLD and AMI patients. Moreover, TYROBP and FCER1G were found to be the common hub genes of the 2 genes through the PPI network, and the common mechanism involved may be immunity and lipid metabolism.^[42]^ TYROBP and FCER1G, important immune-related genes, are both involved in the process of atherosclerosis.^[43,44]^ Abnormal lipid metabolism and atherosclerosis are the physiological and pathological basis of MI, of which both indicate the relationship between MI and immune mechanisms. NEAT1 is a novel lncRNA-type immunomodulator that affects the differentiation and in vivo function of T cells and monocyte-macrophage lineages.^[25]^ Previous studies have shown that NEAT1 is highly expressed in the blood of MI patients and mouse cardiomyocytes. The lncRNA NEAT1 inhibited the expression of ATG12 by suppressing the expression of mir-378a-3p, thus affecting the autophagy of cardiomyocytes.^[25,45]^ A study validating qRT-PCR in 46 MI patients and 40 healthy controls showed a significant increase in KCNQ1OT1 expression in MI patients compared to that in control patients, which is consistent with our findings.^[35]^ Moreover, in MI patients, KCNQ1OT1 expression was shown to be upregulated, while miR-26a-5p expression was downregulated. KCNQ1OT1 knockdown inhibited autophagy and protected cardiomyocytes from apoptosis by upregulating miR-26a-5p.^[46]^ Recent studies suggest that XIST targets the corresponding miRNAs and regulates the pathological processes in MI. Silencing of XIST inhibited myocardial apoptosis in rats with MI by regulating miR-449.^[47]^ XIST promotes cell proliferation and the expression levels of fibrosis-related proteins after MI by sponging miR-155-5p.^[48]^ It can be seen that NEAT1, KCNQ1OT1, XIST, FCER1G, and TYROBP play key roles in the immune regulation of MI, and our findings may lead to novel insights into the pathogenesis of MI immune regulation.

Importantly, our study has certain limitations. First, the small number of original cases in each dataset may have led to false-positive results; therefore we selected 2 datasets for validation and combined them with PCR validation. Second, the study was retrospective, and the appropriate clinical information was lacking. Finally, the exact mechanism by which the ceRNA network regulates the pathogenesis of MI remains unclear. In future studies, we will expand the patient population and use additional in vivo and in vitro samples to confirm the role of these immune-associated lncRNAs in MI and explore their function to further investigate their clinical application value and expression mechanisms.

## 5. Conclusion

In summary, we constructed a lncRNA-mediated MI immune-related ceRNA network using Gene Expression Omnibus transcriptome data, and the key MI-related lncRNAs and hub genes were screened and verified. These important immune-related lncRNAs may play an important role in the pathogenesis and clinical treatment of MI.

## Author contribution

**Conceptualization:** Dongmei Wei.

**Data curation:** Yuanting Meng.

**Funding acquisition:** Hua Fan.

**Project administration:** Hua Fan.

**Software:** Rongtao Chen.

**Writing – original draft:** Yang Sun.

**Writing – review & editing:** Yang Sun.

## Supplementary Material


